# COVID‐19 vaccination in children as a global dilemma through an ethical lens: A retrospective review

**DOI:** 10.1002/hsr2.976

**Published:** 2022-12-03

**Authors:** Masoud Assadi, Mehrzad Kiani, Ehsan Shamsi Gooshki, Zeinab Aryanian, Zeinab M. Afshar, Parvaneh Hatami

**Affiliations:** ^1^ Department of Medical Ethics Shahid Beheshti University of Medical Sciences Tehran Iran; ^2^ Health Research Institute Babol University of Medical Sciences Babol Iran; ^3^ Department of Medical Ethics, Faculty of Medicine/Medical Ethics and History of Medicine Research Center Tehran University of Medical Sciences Tehran Iran; ^4^ Autoimmune Bullous Diseases Research Center Tehran University of Medical Sciences Tehran Iran; ^5^ Department of Dermatology Babol University of Medical Sciences Babol Iran; ^6^ Clinical Research Development Center, Imam Reza, Hospital, Kermanshah University of Medical Sciences Kermanshah Iran

**Keywords:** COVID‐19 vaccine, ethical issues, moral issues, pediatric population

## Abstract

**Background and Aims:**

COVID‐19 pandemic led to a need to rapidly vaccinate as many people as possible. Children are an important part of the population with different characteristics which vaccinating them is a matter of great importance as it should be decided considering all aspects and ethics. Here, we present different aspects of COVID vaccination in children including the potential challenges.

**Methods:**

We searched on PubMed, Google Scholar and Scopus in this regard, and all of the relevant papers published until June 28, 2021 were included if we could access their full‐texts.

**Results:**

We found various expert opinions in this regard and tried to summarized them. Saving lives has similar ethical value as preventing evitable adverse event. Accordingly, mandating the children to receive the SARS‐CoV‐2 vaccine, needs risk‐benefit weighing with special consideration of ethical challenges.

**Conclusion:**

Considering the vast range of benefits resulted from pediatric vaccination both for the children and the community, implementing the program in a scientific manner and also with the least financial expenses for the families seems to be reasonable and makes it both ethical and moral.

## 1 INTRODUCTION

The severe acute respiratory syndrome coronavirus 2 (SARS‐CoV‐2) pandemic has posed a life‐threatening challenge for the world population resulted in an urgent need to rapidly vaccinate as many people as possible.^1^ However, attention needed not to expose the population to excessive risks. Children are an important part of the population with different characteristics and subsequently different needs. Therefore, vaccinating this population is a matter of great importance as it should be decided after considering all aspects and ethics.^2^

In general, the main aim of vaccination is to prevent an infection‐associated morbidity and mortality in the community. Therefore, those individuals with vulnerability to the coronavirus disease 2019 (COVID‐19) complications are prioritized during vaccination program. The elderly (above 65 years of age) and those with comorbidities, such as diabetes, hypertension, cardiovascular diseases, malignancy and obesity, are the population at risk of morbidity and mortality due to the SARS‐CoV‐2 infection.^3^ On the other hand, children are at lower risk of being infected with SARS‐CoV‐2, with lower associated severity, complications and mortality compared with adults.^4^ Hence, children had not been prioritized in conditions with vaccine resources shortage, but with COVID‐19 vaccines becoming more available, lawmakers are thinking out policies to mandate children to be vaccinated to attending schools. Lack of enough data regarding cons and pros of vaccination in children has led to a significant amount of hesitancy in parents for vaccinating their children, thereby bringing about challenges to the ideal vaccination coverage.^5^ There are some practical and ethical questions arise in this regard: what is the role of vaccination in pediatric population? What are limitations and ethical challenges in this universal program?

Here, we present different aspects of pediatric vaccination against SARS‐CoV‐2 with particular focus on ethical considerations.^6^

### 1.1 Where we are on the program of children vaccination against COVID‐19

As it is evident, many countries have initiated the COVID‐19 vaccination program in children.^7^ First, the Pfizer‐BioNTech and Moderna vaccines were approved in children above 12 years of age,^8^ and recently a new formulation of Pfizer‐BioNTech COVID‐19 vaccine for use in children aged 5–11 years has been recommended.^9^ Moreover, testing other vaccines safety for children are underway in different countries.^10^

It is obvious that implementation of vaccination programs to cover the whole population or induce herd immunity is necessary for saving as many lives as possible. Therefore, mandatory children vaccination policies appear to be reasonable.^11^ In general, all governments would agree to mandate the law of vaccinating children against COVID‐19 to achieve herd immunity only if ethical principles are implemented, which include posing the least disturbance on personal rights and freedoms and avoiding the potential harms.^12^

However, getting more close to the details reveals many challenges; vaccine mandating is in fact obliging parents to make decision about their children's vaccination. This issue has many arguments for and against that should be discussed.

Children mandatory vaccination against SARS‐CoV‐2 has been performed in some parts of the world, so as they cannot attend schools if not vaccinated, unless medically contraindicated.^13^ In some countries even financial assistance payments are withheld if families do not comply with children's vaccination program schedule for COVID‐19.^14^ Governments in some parts of the world even consider vaccine refusal as a crime and implement the compulsory vaccination program for children, so as consider imprisoning or fining parents who do not consent to their children's vaccination.

### 1.2 The necessity of pediatric vaccination program against SARS‐CoV‐2

Though the older age and immunosuppressed individuals have been first prioritized for being vaccinated against SARS‐CoV‐2,^15^ children are now the ones at risk of infection, and the resultant complications, hospitalization and mortality.^16^ COVID‐19 has not had significant mortality in children since the beginning of the pandemic; the exception has been the multisystem inflammatory syndrome in children (MIS‐C) that has led to special concerns in the pediatric population affected by the SARS‐CoV‐2 infection.^17^ However, unlike the wild type virus, SARS‐CoV‐2 new variants have affected children more severely, which has been contemplated in thinking out solutions to prevent the severe infections in children.^16^ The Delta variant had led to higher rates of infection in children and young adults, compared with previous variants.^18^ Furthermore, the Omicron variant has caused the most profound risk of morbidity and mortality in pediatrics compared with all the previous emerging SARS‐CoV‐2 variants.^19^ Therefore, preventing even a low number of severe diseases in children by vaccinating them is worth it.

Since keeping the children away from social activities including schools and playgrounds with strict lockdowns has had a significant negative psychological impact on children, COVID‐19 vaccines can be cost‐effective from a societal point of view. However, increasing school outbreaks of infection as the result of viral spread from the infected school children to teachers and other children has been reported with schools reopening which can be prevented by vaccinating children.^20^, ^21^

Another issue is the fact that children tend to be infected with respiratory viruses more frequently with less severe complications and even being asymptomatic,^22^ thereby shed the virus for a relatively long time, making them reservoirs who contribute greatly in the transmission of the virus to other members of the community, including those at higher risk of infection‐associated complications such as the elderly.^23^ Since SARS‐CoV‐2 infection is usually asymptomatic in children, it is not often identified who is infected, and thereby preventive precautions are not applied.^24^ The most important issue regarding children vaccination against SARS‐CoV‐2 is the fact that achieving herd immunity requires the vaccination of 70%–80% of the population, and children account for more than 20% of the community; moreover, COVID‐19 vaccines produce stronger and more durable antibody response in children, compared with the immunity induced by natural infection.^25^

### 1.3 Arguments against SARS‐CoV‐2 vaccination in children

It is well‐known that COVID‐19 is predominantly a silent infection in children.^26^ It is yet unclear whether asymptomatic infected children who have low viral burden can shed the virus efficiently enough to be the main source of transmission.^27^ Assuming that pediatric vaccination is necessary to induce herd immunity, we are not yet sure of the 100% safety and efficacy of any vaccines, including those against COVID‐19 in the elderly and other susceptible individuals.^28^ In fact, expected usefulness, described as the expected benefits minus the expected harms, both for the child and the society should be guaranteed if mandatory pediatric vaccination program is implemented.^29^

On the other hand, children are the population with a possibly long life‐expectancy and an efficient future ahead; although short‐term safety of SARS‐CoV‐2 vaccines has been justified as nonserious in children 5–11 years of age,^9^, ^30^ long‐term complications are not yet revealed and may weigh out the adverse effects of lockdown. Hence, we should have enough knowledge about the safety of each vaccine in various developmental stages, with the aim of not disturbing their growth, development and well‐being.^31^ Some authorities even believe that there is no need to vaccinate children as most of them have already gotten the natural infection. Others believe that COVID‐19 vaccination is futile, and SARS‐CoV‐2 vaccines not only do not have the ability to prevent the disease, but also delay its onset with the vaccine's waning immunity; moreover, it is hypothesized that SARS‐CoV‐2 vaccination might lead to the emergence of immune escape mutations.^32^

In addition, protecting the vulnerable population might not necessarily need children vaccination.^33^ Moreover, if mandatory SARS‐CoV‐2 vaccination program is implemented for pediatrics, we should first afford the required costs and provide sufficient vaccine supply without any economic burden on the families.^34^

### 1.4 Ethical considerations of enrolling children in COVID‐19 vaccination trials

Since pediatrics are usually affected by the SARS‐CoV‐2 infection asymptomatically, parents are not interested to participate their children in COVID‐19 vaccination trials.^35^ The important point is the fact that enrollment of children, especially those under 12 years of age in COVID‐19 vaccination trials is per se an issue of debate, since they do not have the ability to make decisions about participation in research. Therefore, they cannot give informed consent and their volunteerism is not accepted.^36^ Moreover, the long‐term safety of the vaccines is not determined in this population with longer life expectancy. There is insufficient evidence about the safety, efficacy and dosage of SARS‐CoV‐2 vaccines in the pediatrics, potentially exposing them to an unsafe intervention with no specific benefits for this population.^37^ Therefore, conducting vaccination research on children is still a matter of ethical uncertainty and we should initially be sure of vaccines trials suitability for children's age and stage of physiological, emotional, and psychological development.^38^ Accordingly, different guidelines have different opinions; the Council for International Organizations of Medical Sciences (CIOMS) Ethical Guidelines recommend that children and adolescents must be enrolled in medical research unless contraindicated, with considering the safety profile in adults and minimizing the risks in children.^39^ However, the national South African Health Research Ethics guidelines agree to children vaccination only if the results of the research on adults cannot be attributed to children and the risks are at the least.^40^ In fact, vaccine hesitancy among parents, is a serious public health problem in many countries which seems to be driven by various factors including concerns about the risk of COVID‐19, trust in health care system, parents' trust/distrust in “fake news” data, potential vaccine side effects, child's age and parental socioeconomic status.^41^, ^42^, ^43^, ^44^, ^45^, ^46^ Parental willingness towards or refusal to vaccinating their 5 to 12 years old children against COVID‐19 seems to be different in various countries. China, Italy, England, United States, Canada, and Israel reported a relatively high inclination of parents for vaccinating their children, compared to Middle Eastern countries such as Jordan or Saudi Arabia.^41^, ^42^, ^43^, ^47^, ^48^

### 1.5 Ethical implications of mandatory SARS‐CoV‐2 vaccination program in children

As the COVID‐19 vaccine efficacy is considerably higher in children than the older age, vaccinating this population seems to be the best way to create herd immunity and protect the more vulnerable elderly. However, this can be challenging in conditions with vaccine shortages in countries with low sources,^49^ but the issue of great importance is the question whether it is ethical to vaccinate one population to protect another population.^36^ Children are not the ones with the highest need of SARS‐CoV‐2 vaccines, since consequences of the infection are insignificant in this population. Therefore, vaccinating children would impose extra costs and risks on the pediatric population, in favor of the vulnerable population.^50^ An important question in this regard is why not have a vaccine that is efficient enough directly on the vulnerable population that relieves us the need to mandate children to be vaccinated.^51^

Some authorities believe that using children as means to benefit others might be immoral.^52^ However, others believe that this is true only in conditions where it would impose harm or disrespect to the children and treating children as means is morally wrong only if they do not consent to it. Moreover, using children as means to save the lives of many susceptible people can be a measure of humanity.^53^ In addition, considering the fact that COVID‐19 may lead to severe consequences or mortality even in children, vaccination benefits the children per se, since they also get protected against SARS‐CoV‐2 infection.^54^ Regarding ethical issues, every trial or decision‐making should be justified by the four main principles of medical ethics: autonomy, beneficence, nonmaleficence, and justice.^55^ Therefore, we should regard pediatric mandatory vaccination on the basis of these principles.

The first principle, autonomy, points to the humans right to handle their own behavior; therefore, it is acceptable in those individuals with the capacity to choose and accept the consequences of their choices. Autonomy is an important matter of concern in interventions performed on children, as they do not have the capacity to make autonomous decision about health, well‐being and therapeutic or preventive measures and their parents make decisions about their health care and medical interventions, including vaccination. The principle of freedom in making decision is questioned if voluntary vaccination program is turned into a mandatory one. However, according to John Stuart Mill's Harm‐Principle, all humans are free in decision making unless their decisions pose a harmful risk to others or significant threat to public health.^56^ Therefore, it seems that targeting children in vaccination programs does not take their autonomy away. However, older adolescents who have the capacity to make their own decisions would be an exception and an issue to be discussed.^57^

The second principle, beneficence, is described as benefiting others without harming to oneself. Although children develop less severe SARS‐CoV‐2 infection and benefit less from vaccination, it does not mean that COVID‐19 vaccination has no benefit for children. A minority of children become infected severely and even die. This issue is obviously depicted in the current outbreak of Omicron variant which has led to unanticipated rate of COVID‐related hospitalization and death in children.^58^ Therefore, this principle works out in children despite being more practical for adults.^59^

The third principle, non‐maleficence necessitates lack of harm to others. The COVID‐19 vaccines have been proved to have few risks in the adult population. However, nobody can guarantee these vaccines' lack of side effects on the developmental life of a child in the future. The issue is only justified when SARS‐CoV‐2 causes fatal infections in children, the condition in which mandating children to be vaccinated would prevent death, a worse outcome compared with vaccine‐related adverse events.^60^ The current pandemic inducing more severe diseases in children can also justify vaccination according to this ethical principle.

The last principle, justice, means to assure the community that any member, including all children, should have equal opportunity as the adults to access COVID‐19 vaccines.^61^ According to the principle of fairness, as mandatory vaccination programs for children penalize those families that do not conform to the law, there should be rules in turn, to compensate the vaccine‐related serious adverse events and the resultant burden on the child, family and community.^62^ Therefore, if free vaccination is accessible for all children of the world, this principle would be also justified.

### 1.6 Special ethical considerations for adolescents' vaccination against SARS‐CoV‐2 infection

COVID‐19 vaccines have been approved and available for adolescents aged 12–17 years since months ago.^63^ Children below 13 years of age do not have the maturity and ability to decide for their needs, including health care. Nevertheless, the issue is somewhat different for adolescents.^64^ Legally, adolescents have the capacity to give informed consent to getting vaccinated since a great majority of them are mature enough to decide independently and weigh the risks and benefits of vaccination in short‐term and long‐term; however, parents still possess the authority to decide for adolescents' vaccination in some communities.^57^ In general, adolescent maturity is dependent upon several factors such as age, education level, and personal characteristics.^65^ Thus, some of them may have the right of self‐decision making (SDM) over their parents' opposition.^66^ In fact, autonomy is developing in adolescents and many of them are interested in participating in their own health care. Even, some adolescents have comparable capacity for SDM as the adults. Therefore, the law, government and physicians confront challenges in this settings. However, different countries have different rules for the need to adolescents' consent for vaccination. Nonetheless, physicians can have a supportive role in helping them decide, as they can consult, give information and transparent the benefits of being vaccinated against SARS‐CoV‐2, encouraging the adolescent to consent voluntarily.^67^

### 1.7 Moral and religious implications of obligatory children vaccination against SARS‐CoV‐2

With the settlement of mandatory COVID‐19 vaccine law, many people disagreed to this program, as they believe that obligation is in contrast with human rights and violates the morals.^68^ The point is the fact that although religion and morality are on behalf of human rights, they have some reasons to obligate vaccination in children. In fact, people are responsible not only for themselves, but also for their surrounding environment, including the community. To save more lives through vaccination, there should be policies to protect vulnerable population properly. Even the most libertarian philosophers believe that some of the human rights can be restricted for the purpose of harm prevent to others. Liberty has several aspects besides the human rights; the responsibility of an individual to prevent disturbance of economic, social and health rights should not be overlooked.^69^ Therefore, it is both moral and ethical to mandate vaccination in the pediatric population to benefit the community.^70^ Moreover, COVID‐19 vaccination can be mandated in children as a moral obligation, to prevent the potential harms imposed on the child and the family and society surrounding it by being infected.^71^ In fact, the public harm imposed by infectious diseases like SARS‐CoV‐2 infection, which is measured by the number of lives threatened has comparable consequences as personal harm. The important issue is the fact that even harming to oneself is prohibited in some religions and laws.^72^ About the children's right to make decision, again it does not seem to be immoral to vaccinate them without their legal consent, because they also have the right to be protected against the harms of an infectious disease; and this can be carried out by the parents' and the government consent and policies, respectively.^73^ However, harm prevention and the adverse effects of vaccines should be weighed out in every decision making and policy settlement.^74^ On the other hand, research ethics also justifies the vaccination policies beliefs that it is not ethical to prioritize religious liberty over life‐saving trials.^75^

### 1.8 What to do to decrease the parents' hesitancy in vaccinating their children against COVID‐19

Effective communication with the parents and providing enough transparency and information can increase the confidence in the COVID‐19 vaccines, thereby make the community less hesitant in vaccinating their children, so as a great majority of the population voluntarily comply with it, helping the world attain a herd immunity. Physicians and media can have a great role in gaining parents' trust in this settings.^76^ In addition, we can be optimistic about the mandatory pediatric vaccination program by considering the fact that obligatory children vaccination against SARS‐CoV‐2 does not mean to force parents to vaccinate their children, rather implies that only some of their privileges are withheld if they refuse to obey the law.^11^

## 2 CONCLUSION

In general, any choice has its specific consequences. Saving lives has similar ethical value as preventing evitable adverse event. Accordingly, mandating the children to receive the SARS‐CoV‐2 vaccine, needs risk‐benefit weighing with special consideration of ethical challenges. However, considering the vast range of benefits resulted from pediatric vaccination both for the children and the community, implementing the program in a scientific manner and also with the least financial expenses for the families seems to be reasonable and makes it both ethical and moral.

## AUTHOR CONTRIBUTIONS

**Masoud Assadi**: Conceptualization; formal analysis; investigation. **Mehrzad Kiani**: Data curation; investigation; methodology. **Ehsan Shamsi Gooshki**: Resources; supervision; validation. **Zeinab Aryanian**: Investigation; visualization; writing – original draft. **Zeinab M. Afshar**: Investigation; writing – review & editing. **Parvaneh Hatami**: Data curation; investigation; writing – original draft. All authors have read and approved the final version of the manuscript.

## CONFLICT OF INTEREST

The authors declare no conflict of interest.

## ETHICS STATEMENT

This study has been conducted after obtaining the ethical approval of Babol University of Medical Sciences ethics committee. We declare that this study has been done in accordance to Wiley's Best Practice Guidelines on Publishing Ethics and it is has been performed in an ethical and responsible way, with no research misconduct, which includes, but is not limited to data fabrication and falsification, plagiarism, image manipulation, unethical research, biased reporting, authorship abuse, redundant or duplicate publication, and undeclared conflicts of interest.

## TRANSPARENCY STATEMENT

The lead author Zeinab Aryanian, Zeinab Mohseni Afshar, Parvaneh Hatami affirms that this manuscript is an honest, accurate, and transparent account of the study being reported; that no important aspects of the study have been omitted; and that any discrepancies from the study as planned (and, if relevant, registered) have been explained.

## Data Availability

The data sets generated and analyzed during the current study are available from the corresponding author on reasonable request. Corresponding author had full access to all of the data in this study and takes complete responsibility for the integrity of the data and the accuracy of the data analysis.
